# Average semivariance directly yields accurate estimates of the genomic variance in complex trait analyses

**DOI:** 10.1093/g3journal/jkac080

**Published:** 2022-04-20

**Authors:** Mitchell J Feldmann, Hans-Peter Piepho, Steven J Knapp

**Affiliations:** 1 Department of Plant Sciences, University of California, Davis, CA 95616, USA; 2 Biostatistics Unit, Institute of Crop Science, University of Hohenheim, 70593 Stuttgart, Germany

**Keywords:** average semivariance, genomic heritability, genomic variance, genomic relatedness, linear mixed model, genomic best linear unbiased predictor

## Abstract

Many important traits in plants, animals, and microbes are polygenic and challenging to improve through traditional marker-assisted selection. Genomic prediction addresses this by incorporating all genetic data in a mixed model framework. The primary method for predicting breeding values is genomic best linear unbiased prediction, which uses the realized genomic relationship or kinship matrix (**K**) to connect genotype to phenotype. Genomic relationship matrices share information among entries to estimate the observed entries’ genetic values and predict unobserved entries’ genetic values. One of the main parameters of such models is genomic variance ($\sigma_{g}^{2}$), or the variance of a trait associated with a genome-wide sample of DNA polymorphisms, and genomic heritability ($h_{g}^{2}$); however, the seminal papers introducing different forms of **K** often do not discuss their effects on the model estimated variance components despite their importance in genetic research and breeding. Here, we discuss the effect of several standard methods for calculating the genomic relationship matrix on estimates of $\sigma_{g}^{2}$ and $h_{g}^{2}$. With current approaches, we found that the genomic variance tends to be either overestimated or underestimated depending on the scaling and centering applied to the marker matrix (**Z**), the value of the average diagonal element of **K**, and the assortment of alleles and heterozygosity (*H*) in the observed population. Using the average semivariance, we propose a new matrix, $K_{\mathrm{ASV}}$, that directly yields accurate estimates of $\sigma_{g}^{2}$ and $h_{g}^{2}$ in the observed population and produces best linear unbiased predictors equivalent to routine methods in plants and animals.

## Introduction

Linear mixed model (LMM) analyses are routine in the prediction of breeding values in plants and animals (Henderson 1977; VanRaden 2008; Hayes *et al.* 2009; Albrecht *et al.* 2011; Endelman 2011; Crossa *et al.* 2014; Meuwissen *et al.* 2016; Pincot *et al.* 2020; Petrasch *et al.* 2021) and polygenic risk scores in humans (de los Campos *et al.* 2010; Makowsky *et al.* 2011; Lee *et al.* 2012; Dudbridge 2013; Maier *et al.* 2018; Wray *et al.* 2019; Truong *et al.* 2020), partitioning of sources of variance (Searle *et al.* 1992; Lynch and Walsh 1998; Visscher *et al.* 2008; Kang *et al.* 2010; Piepho 2019; Schmidt *et al.* 2019a, 2019b; Feldmann *et al.* 2021), and controlling for confounding effects in genome-wide association studies (GWAS) (Yu *et al.* 2006; Visscher *et al.* 2012; Korte and Farlow 2013; Visscher *et al.* 2017). Genomic prediction approaches are widely applied in the study of complex traits in natural and experimental populations and facilitate the estimation of genomic variance ($\sigma_{g}^{2}$), genomic heritability ($h_{g}^{2}$), and other quantitative, population, and evolutionary genetic parameters (Bulmer *et al.* 1980; Falconer and Mackay 1996; Lynch and Walsh 1998; Meuwissen *et al.* 2001; Bernardo 2002; Hill *et al.* 2008; Van Heerwaarden *et al.* 2008; Crossa *et al.* 2010; de los Campos *et al.* 2015; Huang and Mackay 2016; Lehermeier *et al.* 2017; Noble *et al.* 2019), and has been widely adopted in plant breeding, human genetics, and biology (Habier *et al.* 2007; Goddard and Hayes 2007; Heffner *et al.* 2009; Bloom *et al.* 2013).

Genomic variance ($\sigma_{g}^{2}$)—the variance explained by genome-wide associations between the underlying quantitative trait locus and DNA markers genotyped in the training population—is often estimated in genetic experiments (Visscher *et al.* 2007; Gao *et al.* 2012; Lee *et al.* 2012, 2013; Lipka *et al.* 2014; Rutkoski *et al.* 2014; Kumar *et al.* 2015; Piaskowski *et al.* 2018; Rice and Lipka 2019; Krause *et al.* 2019; Pincot *et al.* 2020; Petrasch *et al.* 2021; Yadav *et al.* 2021) using genomic relationship matrices (GRMs, **K**), which measure the relatedness among entries (Yang *et al.* 2010; Habier *et al.* 2013). The selection of **K** is used directly in solutions to the mixed model equations and is central to estimating the correct variance components in LMM analyses (Henderson 1953; Searle *et al.* 1992; Lynch and Walsh 1998; Mrode 2014). The phenotypic variance–covariance (**V**) is V=G+R, where $R=I\sigma_{\varepsilon }^{2}$ is the residual variance–covariance, and $G=K\sigma_{g}^{2}$ is the genomic variance–covariance (Henderson 1953; Searle *et al.* 1992; Lynch and Walsh 1998; Piepho 2019). The genomic variance $\sigma_{g}^{2}$ is a scalar and, thus, any change in **K** will impact $\sigma_{g}^{2}$ estimates. Genomic variance is found in many ratios throughout modern quantitative genetic research, including genomic heritability, prediction accuracy, selection reliability, prediction error variance, and response to genomic selection (Goddard 2009; Hickey *et al.* 2009; Gorjanc *et al.* 2015). Of these ratios, genomic heritability has been the most frequently reported in public research (Speed *et al.* 2012, 2017; Speed and Balding 2015; de los Campos *et al.* 2015; Legarra 2016; Lehermeier *et al.* 2017; Yang *et al.* 2017).

Genomic heritability is

(1) $$h_{g}^{2}=\frac{\sigma_{g}^{2}}{\sigma_{g}^{2}+\sigma_{\varepsilon }^{2}},$$

where $\sigma_{g}^{2}$ is the genomic variance and $\sigma_{\varepsilon }^{2}$ is the residual variance on an entry-mean basis. Genomic heritability is often estimated by substituting restricted maximum likelihood (REML) variance component estimates into (1). We studied how different forms of **K** affect variance component estimates. We found that, even for large data sets, there are systematic differences in the genomic variance component estimates arising from different forms of **K** (VanRaden 2008; Astle *et al.* 2009; Yang *et al.* 2010; Forni *et al.* 2011; Endelman and Jannink 2012) and that the resulting variance component estimates may not always be correct when directly substituted into (1), as is routine practice. Despite this, researchers often simultaneously use the same approaches for genomic prediction and variance component estimation and may consequently report incorrect genomic heritability estimates.

Here, using the average semivariance (ASV), introduced by Piepho (2019) and expanded by Feldmann *et al.* (2021), we derive a new form for **K**, referred to as $K_{\mathrm{ASV}}$, which is the product $\bar{K}=\bar{Z}\;{\bar{Z}}^{T}$ from the mean-centered marker matrix $\bar{Z}=PZ$, where $P=I_{n}-n^{-1}1_{n}1_{n}^{T}$ is the idempotent, mean-centering *n *×* n*-matrix. The ASV relationship matrix is

(2) $$K_{\mathrm{ASV}}=\frac{\bar{K}}{\left(n-1{)}^{-1}tr(\bar{K}\right)}.$$

This matrix is scaled to the residual variance–covariance matrix and directly yields accurate estimates of $\sigma_{g}^{2}$ and $h_{g}^{2}$ regardless of population constitution, population size, or true heritability. It is possible to scale other forms of the via a division by ${\left(n-1\right)}^{-1}tr(K)$ or to scale estimates of genomic variance from any form of **K** by multiplying ${\left(n-1\right)}^{-1}tr(K)$ by ${\hat{\sigma }}_{g}^{2}$ to obtain ASV estimates of variance component. We explore the practical implications of $K_{\mathrm{ASV}}$ for estimating $\sigma_{g}^{2}$ and $h_{g}^{2}$ in a wild population of *Arabidopsis thaliana* (Atwell *et al.* 2010), a wheat (*Triticum aestivium*) breeding population (Crossa *et al.* 2010), a laboratory mouse (*Mus musculus*) population (Valdar *et al.* 2006), an apple (*Malus × domestica*) breeding population (Kumar *et al.* 2015), and a pig (*Sus scrofa*) breeding population (Cleveland *et al.* 2012). The ASV approach that we propose can be used to estimate variance components in genetic evaluation studies in plants, animals, microbes, and humans.

### The Average Semivariance

The ASV estimator of the total variance (Piepho 2019) is half the average total pairwise variance of a difference between entries and can be decomposed into independent sources of variance, e.g. genomic and residual. There are two alternative ASV derivations, both leading to the same definitions of the estimators. The first derivation originated in geostatistics and estimated the semivariance as half of the variance among all pairwise differences among genotypic values (*g*), i.e. $2^{-1}v\mathrm{ar}(g_{i}-g_{j})$ (Webster and Oliver 2007; Piepho 2019). Piepho (2019) derived ASV from a study’s observations, worked out the semivariance and took the average across all pairs of observations. In our context, there is an equivalent alternative derivation based on the sample variance of the genotypic values Estaghvirou *et al.* (2013). The sample variance among genotypic values is $(n-1{)}^{-1}\sum_{i=1}^{n}(g_{i}-\bar{g}{)}^{2}$. That is to say that the expected values of the sample variance of genotypic values are the ASV, i.e. $E(s_{g}^{2})=\theta_{g}^{\mathrm{ASV}}$. ASV can be used to estimate and partition the total variance in LMM analyses into parts; such as the total variance, as in Piepho (2019), the variance explained by large effect markers and marker–marker interactions, as in Feldmann *et al.* (2021), and genomic variance, as shown below.

### ASV definitions of genomic variance and heritability

In complex traits analyses, there is a crucial difference in the treatment of genotypes and effects in statistical models used for data analysis vs the quantitative genetics theory (Yang *et al.* 2010; Speed *et al.* 2012, 2017; de los Campos *et al.* 2015; Speed and Balding 2015; Legarra 2016). In quantitative genetics theory, between entry differences in genetic values and genomic variance are attributed to the a random sampling of marker genotypes (Bulmer *et al.* 1980; Lande and Thompson 1990; Falconer and Mackay 1996; Lynch and Walsh 1998) and, in an LMM framework, variation stems from a random sampling of the marker effects. Despite differences in derivation and assumptions regarding the source of randomness, the resulting variance–covariance structure between the two coincides under specific experimental, population, and marker sampling conditions (de los Campos *et al.* 2015; Legarra 2016). With this in mind, we derived an approach using the ASV that relies on the assumptions of LMM analyses, e.g. random marker effects, but yields correct estimates of genomic variance.

The analyses shown throughout this paper assume the dependent variables are least squared means (LSMs) or other adjusted means for entries (**y**). $R=I_{n}\sigma_{\varepsilon }^{2}$ gives the residual variance of the LSMs. The ASV can efficiently deal with more general forms of variance–covariance matrices in generalized LMMs (Piepho 2019). The LMM for this analysis is

(3) $$y=1_{n}\mu +I_{n}g+\epsilon$$

where **y** is the vector of phenotypic LSMs of for *n* entries, *n* is the number of entries, $1_{n}$ is an *n*-element vector of ones, *μ* is the population mean, $I_{n}$ is the identity matrix of size *n*, **g** is an *n*-element vector of random effect values for entries with $g∼N(0,K\sigma_{g}^{2})$, and ϵ is the residual for each entry with $\epsilon ∼N(0,I_{n}\sigma_{\epsilon }^{2})$.

The ASV definition of variance from LMM (3) is

(4) $$\theta_{y}^{\mathrm{ASV}}=(n-1{)}^{-1}tr(\mathrm{VP})=\theta_{g}^{\mathrm{ASV}}+\theta_{\varepsilon }^{\mathrm{ASV}},$$

where $\theta_{y}^{\mathrm{ASV}}$ is the phenotypic variance, $V=K\sigma_{g}^{2}+I_{n}\sigma_{\varepsilon }^{2}$ is the variance–covariance among observations, $\theta_{g}^{\mathrm{ASV}}$ is the genomic ASV, and $\theta_{\varepsilon }^{\mathrm{ASV}}$ is the ASV of the residuals. If we assume $G=K\sigma_{g}^{2}$, where **G** is the variance–covariance of the best linear unbiased predictors (BLUPs) of the genotypic values g, it can be inferred that the magnitude of $\sigma_{g}^{2}$ in directly inverse to tr(K) because $V=K\sigma_{g}^{2}+I_{n}\sigma_{\varepsilon }^{2}$.

The ASV definition of the genomic variance is

(5) $$\theta_{g}^{\mathrm{ASV}}=(n-1{)}^{-1}tr(ZZ^{T}P)\sigma_{g}^{2}=(n-1{)}^{-1}tr(\bar{Z}\;{\bar{Z}}^{T})\sigma_{g}^{2}=(n-1{)}^{-1}tr(\bar{K})\sigma_{g}^{2}$$

where $\bar{Z}=PZ$ is the mean-centered marker matrix, and $\bar{K}=\bar{Z}\;{\bar{Z}}^{T}$ is the realized genomic relationship or kinship matrix described by VanRaden (2008), omitting the scaling constant $2\sum_{j}p_{j}(1-p_{j})$, where *p_j_* is the allele frequency of the *j*th SNP, which requires Hardy–Weinberg equilibrium (HWE) to hold (de los Campos *et al.* 2015), and $tr(ZZ^{T}P)=tr(\bar{Z}\;{\bar{Z}}^{T})$. The trace of $\bar{Z}\;{\bar{Z}}^{T}$ is a function of heterozygosity in the observed population (Vitezica *et al.* 2013, 2017; Legarra *et al.* 2018). When the observed population is in HWE, $n^{-1}tr(\bar{K})=1$, and when the population is not in HWE due to inbreeding, the $n^{-1}tr(\bar{K})=1+f$, where *f* is the in coefficient of inbreeding (Endelman and Jannink 2012; Legarra *et al.* 2018). In the general case, $\theta_{g}^{\mathrm{ASV}}=\left(\left(n-1{)}^{-1}tr(\mathrm{KP}\right)\right)\sigma_{g}^{2}$, where **K** is any form of the GRM calculated from **Z**, without centering, or $\bar{Z}$, with centering, because $tr(\bar{K})=tr(KP)$.

The ASV definition of the residual variance is

(6) $$\theta_{\varepsilon }^{\mathrm{ASV}}=(n-1{)}^{-1}\sigma_{\varepsilon }^{2}tr(I_{n}I_{n}^{T}P)=\sigma_{\varepsilon }^{2}.$$

Notably, the genomic variance $\theta_{g}^{\mathrm{ASV}}$ is on the same scale as the residual variance $\theta_{\varepsilon }^{\mathrm{ASV}}$, and both are defined such that (4) is accurate. REML estimates of the residual variance are equivalent to ASV estimates when best linear unbiased estimators or LSMs are the response variable *y*.

### Two equivalent methods yield accurate $h_{g}^{2}$ estimates

There are two equivalent ways to obtain accurate estimates of genomic variance and subsequently genomic heritability. The first method, our recommended approach, utilizes $K_{\mathrm{ASV}}$ (2) in the LMM analysis and directly yields accurate estimates of the genomic variance components from the model by rescaling the GRM. The first method works because $V=K\sigma_{g}^{2}+I_{n}\sigma_{\varepsilon }^{2}$ is a true statement regardless of **K**, but different choices of **K** change the scaling and interpretation of $\sigma_{g}^{2}$. Thus, variance components estimated by ASV can then be substituted directly into (1) without any adjustment.

The second method is to adjust the genomic variance component estimates from any form of **K** by multiplying them by a scaling factor (${\left(n-1\right)}^{-1}tr(\bar{K})$) defined by the population size (*n*) and the diagonals of the chosen GRM ($tr(\bar{K})$). Through substitution of (5) and (6) into (1), the ASV estimator of genomic heritability $h_{g}^{\mathrm{ASV}}$ is

(7) $${\hat{h}}_{g}^{\mathrm{ASV}}=\frac{{\hat{\theta }}_{g}^{\mathrm{ASV}}}{{\hat{\theta }}_{y}^{\mathrm{ASV}}}=\frac{{\hat{\theta }}_{g}^{\mathrm{ASV}}}{{\hat{\theta }}_{g}^{\mathrm{ASV}}+{\hat{\theta }}_{\varepsilon }^{\mathrm{ASV}}}=\frac{(n-1{)}^{-1}tr(\bar{K}){\hat{\sigma }}_{g}^{2}}{(n-1{)}^{-1}tr(\bar{K}){\hat{\sigma }}_{g}^{2}+{\hat{\sigma }}_{\varepsilon }^{2}}.$$

This formulation can be used directly with any form of **K** or $\bar{K}$ by substituting REML variance component estimates. Note that ${\left(n-1\right)}^{-1}tr(\bar{K})$ is the same as the scaling coefficient used in (2). The second strategy is analogous to the post hoc adjustment approach Feldmann *et al.* (2021) proposed.

## Materials and methods

### Genomic relationship matrices

We calculated and applied seven relationship matrices for each population, simulated or case example, including $K_{\mathrm{ASV}}$. We used *AGHmatrix::Gmatrix()* to calculate the Yang *et al.* (2010) ($K_{Y}$) and VanRaden (2008) relationship ($K_{\mathrm{VR}}$) matrices (Rampazo Amadeu *et al.* 2016), *rrBLUP::A.mat()* to calculate the Endelman and Jannink (2012) ($K_{\mathrm{EJ}}$) relationship matrix, and *statgenGWAS::kinship()* to estimate the Astle *et al.* (2009) ($K_{\mathrm{AB}}$) and IBS relationship ($K_{\mathrm{IBS}}$) matrices (van Rossum and Kruijer 2020).

The form proposed by VanRaden (2008) is

(8) $$K_{\mathrm{VR}}=\frac{\bar{Z}\;{\bar{Z}}^{T}}{2\sum_{j=1}^{m}p_{j}(1-p_{j})},$$

where $\bar{Z}$ is the marker matrix centered on column means ($2p_{j}$), and *p_j_* is the minor allele frequency (MAF) for the *j*th SNP. This form assumes HWE and obtains *p_j_* from a historical reference population, not the observed population. When *p_j_* originates from the observed population, the centering by $2p_{j}$ is equivalent to column centering and $K_{\mathrm{VR}}$ only differs from $K_{\mathrm{ASV}}$ by a scaling factor.

The normalized relationship matrix, $K_{GN}$, was explicitly introduced as the normalized relationship matrix by Forni *et al.* (2011) as

(9) $$K_{GN}=\frac{\bar{K}}{n^{-1}tr(\bar{K})}.$$

This form is the most numerically similar to $K_{\mathrm{ASV}}$ and only differs by a single denominator degree of freedom.

The form of the relationship matrix proposed by Endelman and Jannink (2012) is

(10) $$K_{\mathrm{EJ}}=\frac{\delta S_{ii}I+(1-\delta )S+\langle {\bar{Z}}_{•j}\rangle \langle {\bar{Z}}_{•j}^{T}\rangle }{2\langle p_{j}(1-p_{j})\rangle },$$

where $\delta \approx (n/m)CV^{-2}$ is a shrinkage factor, CV^2^ is the coefficient of variation of the eigenvalues of **S**, $S=m^{-1}\bar{Z}\;{\bar{Z}}^{T}-\langle {\bar{Z}}_{•k}\rangle \langle {\bar{Z}}_{•k}^{T}\rangle ,\;\langle S_{ii}\rangle$ is the mean of diagonal elements of **S**. Notably, at high marker densities, when *δ *= 0, Endelman and Jannink (2012) is equivalent to VanRaden (2008).

The method proposed by Yang *et al.* (2010) also centers the columns of **Z** by subtracting $2p_{j}$

(11) $$K_{Y_{ik}}=\{\begin{array}{cc} m^{-1}\sum_{j=1}^{m}\frac{\left(z_{ji}-2p_{j})(z_{jk}-2p_{j}\right)}{2p_{j}(1-p_{j})}, & i\neq k\\ 1+m^{-1}\sum_{j=1}^{m}\frac{z_{ji}^{2}-(1+2p_{j})z_{ji}+2p_{j}^{2}}{2p_{j}(1-p_{j})}, & i=k \end{array},$$

where *z_ij_* is the *j*th SNP in the *i*th individuals, *z_jk_* is the *j*th SNP in the *k*th individual when j≠k, and *m* is the number of markers. The diagonals are treated differently than the off-diagonals in this form.

The method proposed by Astle *et al.* (2009) is

(12) $$K_{\mathrm{AB}}={\left(2m\right)}^{-1}\sum_{j=1}^{m}\frac{(z_{j}-2p_{j}1)(z_{j}-2p_{j}1{)}^{T}}{2p_{j}\left(1-p_{j}\right)},$$

where *z_j_* is the *i*-element vector of the *j*th SNP.

The classical identity-by-state definition is (Astle *et al.* 2009):

(13) $$K_{\mathrm{IBS}}={\left(2m\right)}^{-1}\sum_{j=1}^{m}\left(z_{j}-1\right){\left(z_{j}-1\right)}^{T}+\frac{1}{2}.$$

Note that this is the only calculation that is not scaled or centered by any function of *p_j_*.

For each model and each simulation, we estimated two variance components ($\sigma_{g}^{2}$ and $\sigma_{\varepsilon }^{2}$) using *sommer::mmer()* and took the ratio of variance components in R v4.1.0 (R Core Team 2020). We estimated genomic heritability using the standard form by substituting REML estimates from (3) into (1).

### LMM analysis in R

In the *sommer* R package (Covarrubias-Pazaran 2016), LMM (3) is expressed as

$$\begin{array}{l} m\mathrm{mer}(\mathrm{fixed}=Y\;∼1,\\ r\mathrm{andom}=∼\mathrm{vs}(\text{Entry},\;Gu=K),\\ r\mathrm{cov}=∼\text{units},\\ d\mathrm{ata}=\text{data}) \end{array}$$

where *data* is an n×2 matrix with *Y* as a column of LSMs, *Entry* is a column of factor-coded entries, and *K* is one of the seven GRMs in this study given. A large number of statistical computing solutions can fit this model, including *regress* (Clifford and McCullagh 2006), *ASREML* (Butler 2021), *rrBLUP* (Endelman 2011), *GEMMA* (Zhou and Stephens 2012), *emmREML* (Akdemir and Okeke 2015), *brms* (Bürkner 2017), and *lme4GS* (Caamal-Pat *et al.* 2021).

### Simulated data

We generated 36 experiment designs with different heterozygosity H= 0.0, 0.25, 0.5, and 0.75 and different trait heritability $h_{g}^{2}=$ 0.2, 0.5, and 0.8 and for population sizes of n= 250, 500, and 1,000. In all examples, 1,000 populations genotyped at *m *=* *5,000 causal loci were used to generate the genetic traits. We simulated all *m *=* *5,000 marker effects following a normal distribution *μ* = 0 and *σ* = 1. When multiplied by the marker genotypes and summed, the score is an individual’s true genetic value, *g*. Residuals were simulated with *μ* = 0 and $\sigma_{\varepsilon }^{2}=(1-h^{2})/(h^{2}s_{g}^{2})$ to obtain a trait with the desired genomic heritability (Endelman 2011) and $s_{g}^{2}=(n-1{)}^{-1}\sum_{i=1}^{n}(g_{i}-\bar{g}{)}^{2}$ is the sample variance among genotypic values (Estaghvirou *et al.* 2013). In this study, the true value of $h_{g}^{2}=0.2$, 0.5, or 0.8. All plots were made with the *ggplot2* package (Wickham 2016) in R 4.1.0 (R Core Team 2020).

### Empirical data

We analyzed four publicly available data sets using seven methods for calculating the realized relationship matrix and estimated $h_{g}^{2}$. First, we analyzed six traits from Kumar *et al.* (2015), which evaluated a breeding population of *n *=* *247 apple (*Malus × domestica*) hybrids genotyped at *m *=* *2,829 SNPs with *H *=* *0.348 (Kumar *et al.* 2015). The reported traits were fruit weight (WT), fruit firmness (FF), greasiness (GRE), crispiness (CRI), juiciness (JUI), and flavor intensity (FIN). The shrinkage factor *δ* from Endelman and Jannink (2012) was equal to 0.02. Second, we analyzed the wheat data set from Crossa *et al.* (2010), who evaluated *n *=* *599 wheat (*Triticum aestivum*) fully inbred lines (*H *=* *0.0; δ=0.03) for grain yield (GY) in four environments genotyped for *m *=* *1,278 SNPs. We evaluated each environment (i.e. GY-E1, GY-E2, GY-E3, and GY-E4) with an independent model. Third, we analyzed data from Valdar *et al.* (2006) which evaluated a laboratory population of *n *=* *1,814 stock mice (*M.* *musculus*) for body mass index (BMI), body length, and weight and genotyped for *m *=* *10,346 SNPs (*H *=* *0.363; δ=0.01). Fourth, we analyzed a population of *n *=* *1,057 naturally occurring *Arabidopsis* (*A.* *thaliana*) ecotypes phenotyped for the mean (*μ*) and SD of flowering time under 10°C (FT10) and 16°C (FT16) and genotyped at *m *=* *193,697 SNPs (*H *=* *0.0; δ=0.0) from Atwell *et al.* (2010) and Alonso-Blanco *et al.* (2016). Fifth, we analyzed a commercial pig (*S.* *scrofa*) population made available by PIC (a Genus company) with *n *=* *3,534 entries genotyped at *m *=* *52,843 SNPs (*H *=* *0.311; δ=0.0) that were phenotyped for five traits: T1, T2, T3, T4, and T5 (Cleveland *et al.* 2012). For each population, we calculate the seven relationship matrices (8–9) and apply them in (3) for each trait to estimate ${\hat{h}}_{g}^{2}$ with (1).

We performed cross-validation to determine predictive ability $r(\hat{g},y)$, or the correlation between BLUPs and LSM, which is a measure of success commonly reported in genome prediction studies that indicates how informative the phenotype is as a measure of the genomic value. We also estimated the prediction accuracy $r(\hat{g},y)/\sqrt{{\hat{h}}_{g}^{2}}$, which is a measure of success that scales the predictive ability to the upper limit ($\sqrt{{\hat{h}}_{g}^{2}}$) (Crossa *et al.* 2014). An ideal situation for genomic prediction is a low value of predictive ability and a high value of prediction accuracy. When the predictive ability is high, genomic selection is unlikely to outperform phenotypic selection. When the prediction accuracy is low, the model is bad at capturing the variation in genomic values. We first split each population into 80% train and 20% test and estimated genomic BLUPs and then calculated the accuracy as the correlation between the estimated LSM *y* and the BLUP $\hat{g}$ for all entries in the test set. We performed this cross-validation scheme 100 times for each population and each trait.

## Results

### Analysis of simulated data confirms that ASV yields accurate estimates of genomic variance

**Fig. 1.**
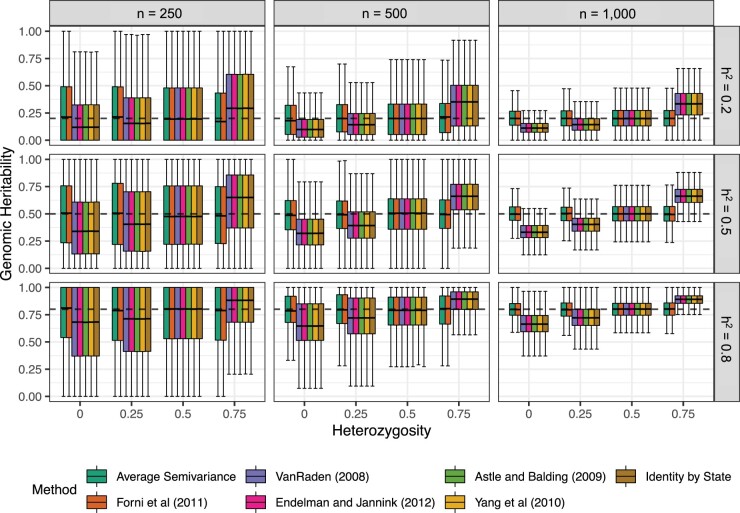
Effect of heritability ($h_{g}^{2}$), population size (*n*), and heterozygosity (*H*) on the accuracy of genomic heritability estimates. Phenotypic observations were simulated for 1,000 samples with *n *=* *250, 500, and 1,000 (left to right) genotyped for *m *=* *5,000 SNPs and the average heterozygosity H= 0%, 25%, 50%, and 75%. The accuracy of genomic heritability estimates (${\hat{h}}_{g}^{2}$) from LMMs fit using the seven relationship matrices is shown for true genomic heritability ($h_{g}^{2}$) =0.2 (upper panel), 0.5 (middle panel), and 0.8 (lower panel). The upper and lower halves of each box correspond to the first and third quartiles (the 25th and 75th percentiles). The notch corresponds to the median (the 50th percentile). The upper whisker extends from the box to the highest value that is within 1.5 IQR of the third quartile, where IQR is the interquartile range, or distance between the first and third quartiles. The lower whisker extends from the first quartile to the lowest value within 1.5 IQR of the quartile. The dashed line in each plot is the true value from simulations.

The ASV relationship matrix yielded suitable estimates of genomic variance and genomic heritability in the observed populations, while the other methods varied with the level of heterozygosity. When heterozygosity *H *<* *0.5, the genomic variance tends to be underestimated, and when *H *>* *0.5, the genomic variance tends to be overestimated (Fig. 1) by methods excluding (2) and (9). This pattern was realized regardless of the population size, e.g. n= 250, 500, and 1,000. All methods tend to produce accurate estimates when *H *=* *0.5, in which case the inbreeding coefficient *f *=* *0 and HWE is not violated.

**Fig. 2.**
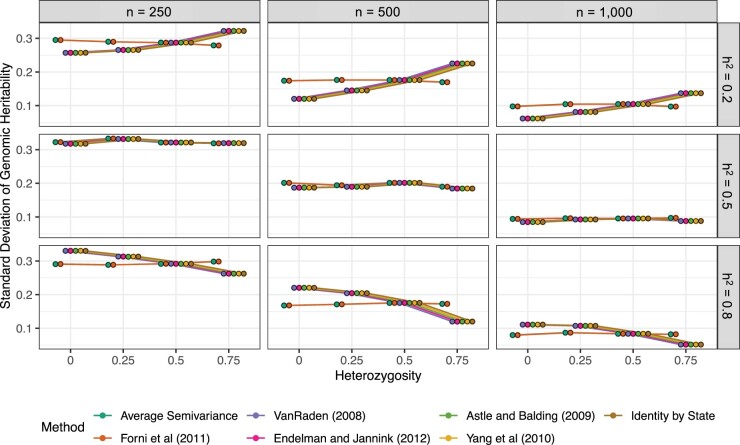
Precision of genomic heritability estimates from simulations. The SDs from the simulation experiments are plotted against heterozygosity (*H*), population size (*n*), and true genomic heritability ($h_{g}^{2}$) for each of the seven GRMs evaluated in this study. Points and lines are jittered around each value of *H* to improve clarity as many of the lines are parallel and overlap one another.

The precision (variance) improved by increasing the population size (*n*), but the accuracy (bias) did not improve. It has been demonstrated ad nauseam that increasing *n* increases precision or lowers the sampling variance of the estimates but does not eliminate bias (Laird and Ware 1982; Searle *et al.* 1992; Lynch and Walsh 1998; Legarra 2016). Notably, the entire parameter space of $h_{g}^{2}$ was observed when the population size is small (Fig. 1). Only $K_{\mathrm{ASV}}$ and $K_{\mathrm{GN}}$ yielded stable precision as *H* increased (Fig. 2). Other methods that we examined have variable precision and variable accuracy depending on the sample size, heterozygosity, and the true value of $h_{g}^{2}$ (Figs. 1 and 2). Interestingly, we observed an interaction between $h_{g}^{2}$ and *H* that impacted the precision of genomic heritability estimation did not affect $K_{\mathrm{GN}}$ or $K_{\mathrm{ASV}}$. Precision improved as *H* increased for high heritability traits and precision worsened as *H* increased for low heritability traits. For traits where $h_{h}^{2}=0.5$, precision was constant.

### Analysis of simulated and empirical data confirms that ASV does not impact BLUPs or prediction accuracy

**Fig. 3.**
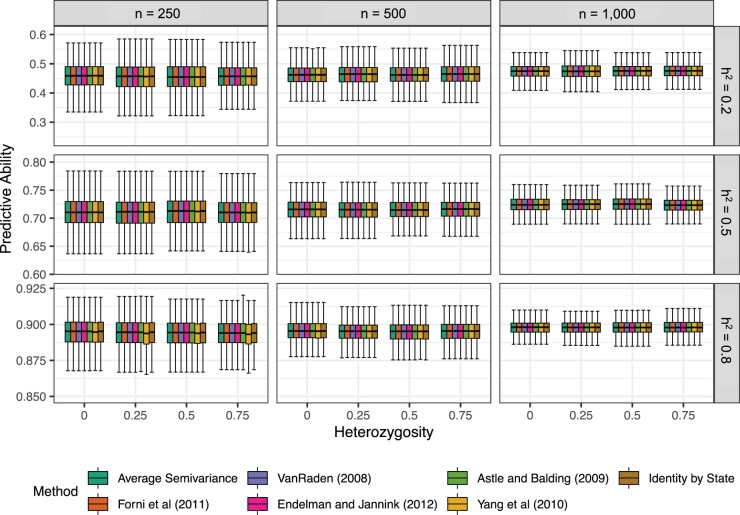
Effect of heritability ($h_{g}^{2}$), population size (*n*), and heterozygosity (*H*) on the predictive ability $r(\hat{g},y)$. Phenotypic observations were simulated for 1,000 samples with *n *=* *250, 500, and 1000 (left to right) genotyped for *m *=* *5,000 SNPs and the average heterozygosity H= 0%, 25%, 50%, and 75%. $r(\hat{g},g)$ estimates from LMMs fit using the seven relationship matrices is shown for true genomic heritability $h_{g}^{2}=0.2$ (upper panel), 0.5 (middle panel), and 0.8 (lower panel). Each box’s upper and lower halves correspond to the first and third quartiles (the 25th and 75th percentiles). The notch corresponds to the median (the 50th percentile). The upper whisker extends from the box to the highest value within 1.5 IQR of the third quartile, where IQR is the interquartile range or distance between the first and third quartiles. The lower whisker extends from the first quartile to the lowest value within 1.5 IQR of the quartile.

**Fig. 4.**
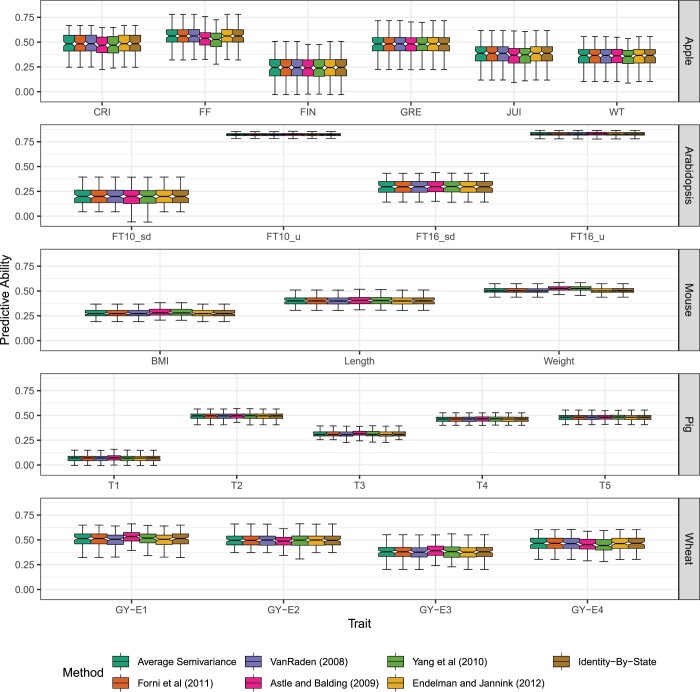
Cross-validated predictive ability from five case studies and including 22 phenotypic traits using seven GRMs. Cross-validated predictive ability ($r(\hat{g},y)$) results are presented from 100 realizations of 80:20 cross-validation using the seven relationship matrices for six traits in an apple population with *n *=* *247 entries genotyped at *m *=* *2,829 SNPs (Kumar *et al.* 2015) (first row), four traits in an *Arabidopsis* population with *n *=* *1,057 entries genotyped at *m *=* *193,697 SNPs (Atwell *et al.* 2010) (second row), three traits in an mouse population with *n *=* *1,814 entries genotyped at *m *=* *10,346 SNPs (Valdar *et al.* 2006) (third row), and five traits in a pig population with *n *=* *3,534 entries genotyped at 52,843 SNPs (Cleveland *et al.* 2012) (fourth row), four traits in an wheat population with *n *=* *599 entries genotyped at *m *=* *1,278 SNPs (Crossa *et al.* 2010) (fifth row). For the *Arabidopsis* data set (second row), $K_{Y}$ systematically produced singular systems in *sommer::mmer()* and prediction accuracy was not estimated for either $FT10_{\mu }$ or $FT16_{\mu }$. Each box’s upper and lower halves correspond to the first and third quartiles (the 25th and 75th percentiles). The notch corresponds to the median (the 50th percentile). The upper whisker extends from the box to the highest value within 1.5 IQR of the third quartile, where IQR is the interquartile range or distance between the first and third quartiles. The lower whisker extends from the first quartile to the lowest value within 1.5 IQR of the quartile.

**Fig. 5.**
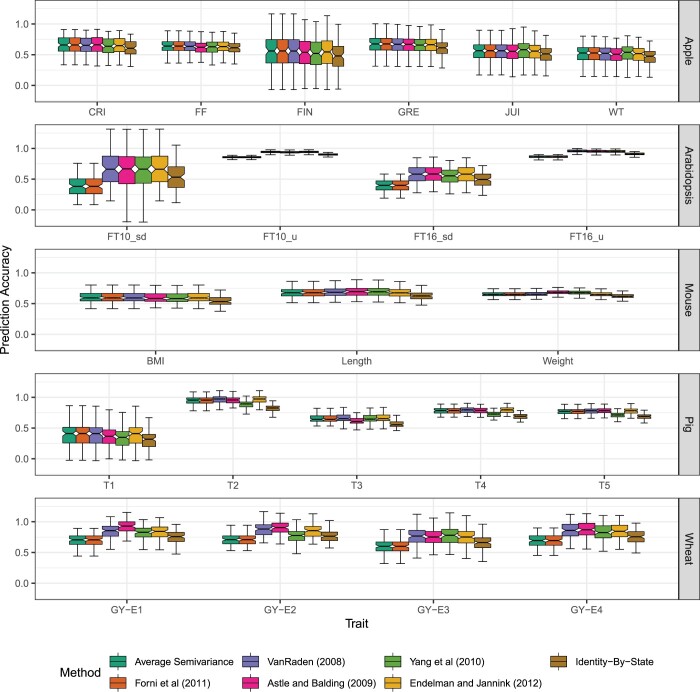
Cross-validated prediction accuracy from five case studies and including 22 phenotypic traits using seven GRMs. Cross-validated prediction accuracy ($r(\hat{g},y)/\sqrt{{\hat{h}}^{2}}$) results are presented from 100 realizations of 80:20 cross-validation using the seven relationship matrices for six traits in an apple population with *n *=* *247 entries genotyped at *m *=* *2,829 SNPs (Kumar *et al.* 2015) (first row), four traits in an *Arabidopsis* population with *n *=* *1,057 entries genotyped at *m *=* *193,697 SNPs (Atwell *et al.* 2010) (second row), three traits in an mouse population with *n *=* *1,814 entries genotyped at *m *=* *10,346 SNPs (Valdar *et al.* 2006) (third row), and five traits in a pig population with *n *=* *3,534 entries genotyped at 52,843 SNPs (Cleveland *et al.* 2012) (fourth row), four traits in an wheat population with *n *=* *599 entries genotyped at *m *=* *1,278 SNPs (Crossa *et al.* 2010) (fifth row). For the *Arabidopsis* data set (second row), $K_{Y}$ systematically produced singular systems in *sommer::mmer()* and prediction accuracy was not estimated for either $FT10_{\mu }$ or $FT16_{\mu }$. Each box’s upper and lower halves correspond to the first and third quartiles (the 25th and 75th percentiles). The notch corresponds to the median (the 50th percentile). The upper whisker extends from the box to the highest value within 1.5 IQR of the third quartile, where IQR is the interquartile range or distance between the first and third quartiles. The lower whisker extends from the first quartile to the lowest value within 1.5 IQR of the quartile.

Neither the predictive ability ($r(\hat{g},y)$) nor the BLUPs from genomic best linear unbiased predictor are affected by ASV. In our simulated populations, the predictive ability was equal across all seven GRMs that we tested (Fig. 3), but the prediction accuracy (${\hat{h}}_{g}^{-1}r(\hat{g},y)$) varies with the choice of GRM and therefore the heterozygosity in the sampled populations. In 22 empirical trait × population examples we evaluated, the differences in the prediction accuracy, when present, appeared to be negligible and do not lend themselves clearly to “better” or “worse” categories (Figs. 4 and 5). While the choice of **K** does not impact BLUP, it does impact estimates of genomic variance ${\hat{\sigma }}_{g}^{2}$, genomic heritability ${\hat{h}}_{g}^{2}$, prediction accuracy ${\hat{h}}_{g}^{-1}r(\hat{g},y)$ (Fig. 5), average prediction error variance PEV, and selection reliability $1-\sigma_{g}^{-2}P\mathrm{EV}$, which all rely on ${\hat{\sigma }}_{g}^{2}$. Differences in Fig. 5 are more pronounced for the fully inbred populations, e.g. *Arabidopsis* and wheat, than the partially inbred populations, e.g. pig, mouse, and apple. ASV allows users to understand how well GS is performing relative to phenotypic selection and to predict how reliable genomic selection can be for certain traits in specific populations more accurately than other methods since it directly yields accurate estimates of $\sigma_{g}^{2}$ and $h_{g}^{2}$ (Figs. 3–5).

### The relationship between $K_{\mathrm{ASV}}$ and $K_{\mathrm{GN}}$

We found that the normalized **K**, i.e. $K_{\mathrm{GN}}$ (9), proposed by Forni *et al.* (2011) and further described by Legarra (2016), yields estimates of **K** that only deviated from $K_{\mathrm{ASV}}$ by a single degree of freedom in the denominator of the matrix scaling factor. Although these estimators were derived through different approaches and with different concepts in mind, they are numerically similar, apart from a single degree of freedom difference in the divisor of the GRM: Forni *et al.* (2011) used the number of entries (*n*), whereas we used $df_{g}=n-1$ for calculating the sample variance (Bulmer 1979). $K_{\mathrm{GN}}$, instead of being biased by a factor of $1/(1+f),\;K_{\mathrm{GN}}$ is biased by a factor of (n−1)/n. Our simulations confirm this deviation and the median genomic variance estimates using $K_{\mathrm{GN}}$ were slightly larger than $K_{\mathrm{ASV}}$, which was equal to the true value in the simulations (Fig. 1). This work, Forni *et al.* (2011), and Legarra (2016) all arrive at numerically similar solutions through conceptually different derivations, which we feel is indicative of the value of these approaches for the plant, animal, and human genetic studies that rely on genomic relatedness, e.g. GWAS, genomic prediction, or inferring population structure and ancestry.

### $K_{\mathrm{ASV}}$ yields genomic variance estimates that naturally account for inbreeding

Inbreeding changes the patterns of among and within entry genomic variance and drives deviations from HWE (Bernardo 2002; Wricke and Weber 2010; Legarra 2016; Isik *et al.* 2017). A challenge of partial inbreeding is that researchers may not know or infer the reference population, making unadjusted genomic variance estimates hard to interpret (Legarra 2016). In genomic evaluations in plants and animals, the current population is often interpreted as the reference population, but this is an inaccurate interpretation unless the population is at HWE and *H *=* *0.5 by design or happenstance. It may be that the only reference population that is concretely defined is the sample population. In connection to Legarra (2016), our work will allow researchers to directly obtain accurate estimates of the genomic variance in the sample population regardless of whether the assumptions of HWE are met.

When the study populations are entirely, or partially, inbred as in wheat, *Arabidopsis*, or inbred per se evaluations in hybrid crops, such as maize, tomato, rice, the covariance among marker effects increases. Lehermeier *et al.* (2017) proposed a novel method (termed method M2) to account for the covariance of marker effects, which increases the genomic variance estimates in recombinant inbred line populations. Our analyses of the same flowering time data with ASV yielded equivalent results and patterns to Lehermeier *et al.* (2017), suggesting that $K_{\mathrm{ASV}}$ may be providing an estimate of genomic variance that naturally accounts for linkage disequilibrium (LD) and the covariance of marker effects (Table 1). We believe that the similarity in results is because LD is associated with the off-diagonal elements of **K**, which is taken into account using ASV.

**Table 1.** Genomic heritability (${\hat{h}}_{g}^{2}$) estimates for the 22 traits from five case studies, including six traits in an apple population with *n *=* *247 entries genotyped at *m *=* *2,829 SNPs (Kumar *et al.* 2015), four traits in an wheat population with *n *=* *599 entries genotyped at *m *=* *1,278 SNPs (Crossa *et al.* 2010), four traits in an *Arabidopsis* population with *n *=* *1,057 entries genotyped at *m *=* *193,697 SNPs (Atwell *et al.* 2010), and three traits in an mouse population with *n *=* *1,814 entries genotyped at *m *=* *10,346 SNPs (Valdar *et al.* 2006), and five traits in a pig population with *n *=* *3,534 entries genotyped at 52,843 SNPs (Cleveland *et al.* 2012) using the seven GRMs compared in this article.

Case study	Trait	ASV	Forni et al. (2011)	VanRaden (2008)	Astle and Balding (2009)	Yang *et al.* (2010)	Endelman and Jannink (2012)	IBS
Apple	WT	0.48	0.48	0.49	0.51	0.44	0.50	0.59
	GRE	0.51	0.51	0.52	0.52	0.53	0.53	0.62
	FF	0.77	0.77	0.78	0.75	0.70	0.79	0.84
	CRI	0.54	0.54	0.55	0.50	0.54	0.56	0.64
	JUI	0.47	0.47	0.47	0.44	0.41	0.48	0.57
	FIN	0.19	0.19	0.19	0.20	0.21	0.20	0.26
*Arabidopsis*	FT10${}_{\mu }$	0.92	0.92	0.76	0.77	–	0.76	0.83
	FT10${}_{\text{sd}}$	0.27	0.27	0.09	0.09	0.09	0.09	0.14
	FT16${}_{\mu }$	0.92	0.92	0.75	0.76	–	0.76	0.83
	FT16${}_{\text{sd}}$	0.55	0.55	0.26	0.26	0.29	0.26	0.36
Mouse	BMI	0.21	0.21	0.21	0.23	0.23	0.21	0.26
	Length	0.35	0.35	0.34	0.34	0.34	0.35	0.41
	Weight	0.60	0.60	0.59	0.59	0.60	0.60	0.66
Pig	T1	0.03	0.03	0.03	0.04	0.04	0.03	0.05
	T2	0.27	0.27	0.26	0.27	0.31	0.26	0.36
	T3	0.23	0.23	0.22	0.27	0.23	0.22	0.31
	T4	0.35	0.35	0.34	0.35	0.41	0.34	0.45
	T5	0.39	0.39	0.38	0.38	0.46	0.38	0.49
Wheat	GY-E1	0.53	0.53	0.35	0.33	0.39	0.36	0.46
	GY-E2	0.49	0.49	0.32	0.29	0.41	0.34	0.42
	GY-E3	0.40	0.40	0.24	0.27	0.24	0.25	0.33
	GY-E4	0.45	0.45	0.29	0.27	0.29	0.30	0.38

## Discussion

GRMs are routine in human, plant, animal, and microbial genetics in agriculture, medicine, and biology for both prediction of genetic values, e.g. breeding values and polygenic scores (Hayes *et al.* 2010; Jensen *et al.* 2012; Bloom *et al.* 2013; Gowda *et al.* 2014; Lipka *et al.* 2014, 2015; Goddard *et al.* 2016; Jivanji *et al.* 2019; Pincot *et al.* 2020; Petrasch *et al.* 2021; Fan *et al.* 2021), and for accounting for population structure and relatedness in marker-trait association analyses (Kang *et al.* 2010; Yang *et al.* 2010, 2011; Tian *et al.* 2011; Peiffer *et al.* 2014; Spindel *et al.* 2016; Alqudah *et al.* 2016; Pincot *et al.* 2018; Ferguson *et al.* 2021; Freebern *et al.* 2020). As advocated by Speed and Balding (2015) and Legarra (2016), the ragged diagonal elements of $K_{\mathrm{ASV}}$ equal 1, on average, and the off-diagonal elements equal 0, on average. ASV directly yields accurate estimates of genomic heritability in the observed population and can be used to adjust deviations that arise from other commonly used methods for calculating genomic relationships regardless of the population constitution, such as inbred lines and F_1_ hybrids, unstructured GWAS populations, and animal herds or flocks (Fig. 1).

The interpretation of genomic variance and heritability estimates was systematically affected by the available methods used to estimate **K**. The bias that we show in this paper is independent of sampling error (large data sets mitigate sampling error) and exists even for enormous data sets. We derived a new relationship matrix, $K_{\mathrm{ASV}}$, using the ASV that yielded consistent variance component estimates. We also derived a correction factor ${\left(n-1\right)}^{-1}tr(\bar{K})$ that allowed accurate estimates of genomic heritability in the observed population from LMM analyses using various software packages (Clifford and McCullagh 2006; Endelman 2011; Zhou and Stephens 2012; Pérez and de Los Campos 2014; Akdemir and Okeke 2015; Covarrubias-Pazaran 2016; Bürkner 2017; Runcie and Crawford 2019; Butler 2021; Caamal-Pat *et al.* 2021).

Adopting experiment designs that enable screening of a greater number of entries *n* yield more precise estimates of key variance components in research programs (Smith *et al.* 2006; Moehring *et al.* 2014; Borges *et al.* 2019; Mackay *et al.* 2019; Hoefler *et al.* 2020) and ASV can ensure that those estimates are accurate and comparable across populations. In many plant quantitative genetic studies, the population sizes are n≈500, which may pose a general problem for variance component and ratio estimation as those variance components can have high sampling variability between replicated experiments (Fig. 1). For large populations, common in human and domesticated animal studies, it is possible to precise (low variance) but inaccurate (high bias) estimates of $\sigma_{g}^{2}$ and $h_{g}^{2}$ resulting from different relationship matrices, unless the assumptions of HWE happen to be perfectly met in the study population.

We did not explore differences that arise from population structure or rare alleles, which is a limitation to our simulation approach (Astle *et al.* 2009; Lee *et al.* 2012, 2013; Speed *et al.* 2012). We believe, but have not demonstrated, that our ASV approach could be applied to many of the existing methods that have been proposed to handle these real-world situations. For example, Lee *et al.* (2012) propose that **K** be calculated among different sets of SNPs with similar MAFs and then the genomic variance for each MAF bin are jointly estimated and summed to account for unique variation attributable to common vs rare alleles. Speed *et al.* (2012) proposed a scaling factor for each SNP based on its own sample variance ($\mathrm{var}{\left(x_{l}\right)}^{s}$), where *s* ranges from −2 to 2 and *x_l_* is a vector of marker genotypes at the *l*th locus (Speed *et al.* 2012; Lee *et al.* 2013). This means that SNPs are either being centered and scaled (*s* = –1), which is equal to $K_{\mathrm{GN}}$, or that SNPs are being centered but not scaled (*s *=* *0). While Speed *et al.* (2012) indicate that *s* = –1 yields more stable estimates of $h_{g}^{2}$, it is not entirely clear how to optimally select a value of *s* for each locus.

Our simulations exposed systematic differences between (2) and other forms of **K**. Our simulation and empirical experiments also suggested limited, if any, differences between genomic variance estimates from five other commonly cited GRMs (Fig. 1; Table 1). The lack of significant differences is perturbing. In every case, there are multiple reasons given for using one relationship matrix over any other that do not seem to play any role in either bias (accuracy) or variance (precision) of the genomic variance component estimates. Both (2) and (9) have the necessary numeric properties advocated by Speed and Balding (2015) that enable the variance components from LMM (3) to be interpreted directly as the genomic variance in the sampled population. We recommend that the ASV approach be considered for adoption by genetic researchers working in humans, microbes, or (un)domesticated plants and animals.

## Data availability

The input and output data from simulations and analyses have been deposited, along with the code for the simulations, in a public Zenodo repository (https://doi.org/10.5281/zenodo.6211739).
